# Exploring the Subcellular Localization of *Monascus* Pigments Biosynthases: Preliminary Unraveling of the Compartmentalization Mechanism

**DOI:** 10.3390/jof10060375

**Published:** 2024-05-24

**Authors:** Fei Xiong, Jingyi Wei, Youxiang Zhou, Yanchun Shao, Jiao Liu, Fusheng Chen

**Affiliations:** 1 National Key Laboratory of Agricultural Microbiology, Hubei International Scientific and Technological Cooperation Base of Traditional Fermented Foods, College of Food Science and Technology, Huazhong Agricultural University, Wuhan 430070, China; 2 Hubei Key Laboratory of Nutritional Quality and Safety of Agro-Products, Institute of Quality Standard and Testing Technology for Agro-Products, Hubei Academy of Agricultural Sciences, Wuhan 430064, China

**Keywords:** *Monascus* spp., *Monascus* pigments, *Monascus* pigments biosynthetic proteins, subcellular localization, compartmentalization

## Abstract

*Monascus* pigments (MPs), a class of secondary metabolites produced by *Monascus* spp., can be classified into yellow, orange, and red MPs according to their differences in the wavelength of the maximum absorption. However, the biosynthetic sequence and cellular biosynthesis mechanism of different MPs components are still not yet completely clear in *Monascus* spp. In this study, the subcellular localization of five MPs synthases was investigated using fluorescent protein fusion expression. The results revealed that the proteins encoded by the MPs biosynthetic gene cluster were compartmentalized in various subcellular locations, including the mitochondrial polyketide synthase MrPigA, cytosolic enzymes consisting of the ketoreductase MrPigC, the oxidoreductase MrPigE, and the monooxygenase MrPigN, and the cell-wall-bound oxidoreductase MrPigF. Moreover, the correct localization of MrPigF to the cell wall was crucial for the synthesis of orange MPs. Lastly, we discussed the compartmentalized biosynthetic pathway of MPs. This study will not only be helpful in clarifying the biosynthetic sequence and biosynthesis mechanism of different MPs but also provides new insights into the cellular biosynthesis of secondary metabolites in filamentous fungi.

## 1. Introduction

*Monascus* spp., a genus of filamentous fungi, have been widely utilized in the fermentation, food, and pharmaceutical industries for centuries [1,2], and they are considered as a valuable reservoir capable of generating various secondary metabolites (SMs) with biological activities, including *Monascus* pigments (MPs), monacolin K, and γ-aminobutyric acid [1,3,4]. Among them, MPs, a mixture of pigments with an azaphilone skeleton, can be classified as yellow (YMPs, 330–450 nm), orange (OMPs, 460–480 nm), and red (RMPs, 490–530 nm) pigments based on the variance in the wavelength of the maximum absorption [5]. MPs are best known for being used as food colorants because of their vibrant colors, great solubility, and high safety [3]. In China, the earliest records describing MPs as food colorants can be traced back more than two thousand years [1,6]. Nowadays, MPs are added to a wide variety of foods, such as sausages, bakery foods, and cured meat products, to enhance their color, improve their quality, and prolong their shelf-life [7]. Moreover, MPs also exhibit promising biological and therapeutic activities. For example, YMPs possess the characteristics of reducing blood lipids, anti-obesity properties, and anti-inflammation properties [8,9]; OMPs show great antimicrobial and antitumor activities [10,11,12]; and RMPs display a strong inhibitory effect on cancerous MKN-28 cells [13].

Due to the characteristics of highly complex mixtures, single-component MPs with high purity are impossible to obtain on a large scale [2,14], which not only hinders the utilization of single-component MPs but also limits the development of relevant industries [2]. Therefore, a comprehensive understanding of the biosynthetic pathway of MPs is crucial for constructing efficient single-component production methods. Chen et al. propose the biosynthetic pathway of MPs through gene knockout and heterologous expression in *M. ruber* M7 (Figure S1), which has laid a good foundation for understanding the biosynthetic mechanism of MPs [5]. In detail, the *mrpigA* (polyketide synthases gene) and *mrpigN* (FAD-dependent monooxygenase gene) deletion mutants are unable to produce MPs [15,16]. The gene *mrpigC*, encoding a C-11-keto reductase, is related to the formation of MPs intermediates, namely, M7PKS-1 [5,17]. The *mrpigE* deletion strain loses the ability to yield OMPs [17,18]. In addition, in vitro and in vivo experiments demonstrate that MrPigE is responsible for converting OMPs (rubropunctatin, monascorubrin) into YMPs (monascin, ankaflavin) [19]. The gene *mrpigF*, encoding an FAD-dependent oxidoreductase in the final step of MPs synthesis, is directly responsible for yielding OMPs [5,20]. However, the biosynthetic sequence and cellular biosynthesis mechanism of different MPs components are still not yet completely clear.

Recent studies have revealed that SMs synthesis in filamentous fungal cells is similar to that in plants, showing a highly ordered compartmentalized biosynthesis. The enzymes and specific intermediates required for SMs synthesis are distributed in organelles or cellular compartments such as peroxisomes, endoplasmic reticulum, vesicles, endosomes, etc., thus forming an orderly partitioning for SMs biosynthesis at a cellular level [21,22,23]. This mechanism is vital for fungi to prevent unnecessary metabolic crosstalk, enhance pathway efficiency, and isolate toxic substances [21]. In *Botrytis cinerea*, key enzymes involved in melanin biosynthesis are encapsulated in different cellular compartments to improve enzymatic efficiency and prevent toxic intermediates [24]. Zhang et al. confirmed that the enzymes that participate in mycophenolic acid biosynthesis were compartmentalized, which is significant for understanding the organelle-associated catalytic mechanisms of some SMs in fungi [25]. Lim et al. reported that FmqD, an oxidoreductase in fumiquinazoline (Fq) biosynthesis, was localized to the cell wall to yield fumiquinazoline C (FqC) in *Aspergillus fumigatus*, and the incorrect localization of FmqD in the cytoplasm reduced the FqC yield [26]. These studies have demonstrated that highly ordered compartmentalized assembly lines play important roles in the biosynthesis of fungal SMs.

In this study, we developed a novel and feasible method for the subcellular localization of proteins in *M. ruber* M7 by eliminating the interference of spontaneous fluorescence. Subsequently, the subcellular localization of five key MPs synthases were analyzed by this method. Furthermore, we found that the correct localization of MrPigF to the cell wall was crucial for the synthesis of OMPs. Finally, we discussed the compartmentalized biosynthetic pathway of MPs. Our results provide a valuable insight into the compartmentalized synthetic pathways of MPs, laying a theoretical foundation for producing single-component MPs with a high purity.

## 2. Materials and Methods

### 2.1. Strains and Culture Conditions

*M. ruber* M7 was deposited in the Culture Collection of State Key Laboratory of Agricultural Microbiology (CCAM) (Wuhan, China) with the deposition number CCAM 070120. The *M. ruber* M7 and its recombinant strains used in this study are listed in Table 1 and were cultivated on potato dextrose agar (PDA) slants at 28 °C. *Escherichia coli* DH5α, serving as the plasmid construction host, was cultivated in Luria–Bertani (LB) medium (1% tryptone, 0.5% yeast extract, 1% NaCl, 1 L distilled water) at 37 °C for 12–24 h. *Agrobacterium tumefaciens* EHA105, which was used to obtain the transformants of the *M. ruber* strains, was incubated in LB medium at 28 °C for 36–72 h. PDA media were prepared as previously described for colonial morphologies [27].

### 2.2. Characterization of MPs-Free Mutants

#### 2.2.1. Colonial Morphologies and Fluorescence Signal Observation

First, 2 μL samples of fresh spore suspension (10^5^ spores/mL) of *M. ruber* M7, ∆*mrpigA*+*lig4*+*G*::*G*, and ∆*mrpigN* strains were incubated on PDA media and cultivated at 28 °C for 7 days for observation of colonial morphologies. Then, their mycelia were subjected to full-wavelength fluorescence scanning at 405 nm, 488 nm, and 561 nm using a confocal laser scanning microscope (FV3000, Olympus, Tokyo, Japan). The images were processed with Olympus FV31S-DT and ImageJ [29]. Their fluorescence signals were detected using the DAPI (excitation wavelength: 405 nm, emission wavelength: 430–470 nm), EGFP (excitation wavelength: 488 nm, emission wavelength: 500–540 nm), and mCherry (excitation wavelength: 561 nm, emission wavelength: 570–670 nm) channels. 

#### 2.2.2. Characterization of MPs Biosynthesis Gene

The mycelia of ∆*mrpigA*+*lig4*+*G*::*G* after cultivation on PDA media were collected for RNA extraction. The transcriptional levels of each gene in the MPs biosynthesis gene cluster were analyzed using reverse transcription PCR (RT-PCR). The primers are listed in Table S1. 

To explore whether the downstream enzymes involved in the MPs biosynthesis of the ∆*mrpigA*+*lig4*+*G*::*G* strain can function normally, we conducted feeding experiments using M7PKS-1 (6-(4-hydroxy-2-oxopentyl)-3-methyl-2,4-dioxocyclohexane carbaldehyde), which is the first stable intermediate in MPs biosynthesis [16]. Briefly, 100 mg of M7PKS-1 was dissolved in 2 mL of acetonitrile (ACN) solution, and it was then filtered using a 0.22 μm filter for sterilization. After that, 2 mL of sterile M7PKS-1 solution and ACN solution were fully mixed with 100 mL of PDA media, respectively. A total of 2 μL of spore suspension (10^5^ spores/mL) was inoculated in PDA, PDA+ACN, and PDA+M7PKS-1 media, respectively, and cultivated at 28 °C for 7 d. The mycelia were collected and lyophilized for the extraction and detection of MPs.

### 2.3. Construction and Localization Analysis of EGFP-Labeled Mutants

In order to localize the MrPigA, MrPigC, MrPigN, MrPigE, and MrPigF in vivo, fusion expression vectors containing the target genes and fluorescence-labeled gene were constructed. Firstly, *PgpdA* (promoter), *egfp* (fluorescence-labeled), *TtrpC* (terminator), and *neo* (*neo*mycin-resistant gene) fragments were ligated to pCAMBIA3300 digested with *Kpn*I and *Pst*I using the Gibson assembly technology [30] to obtain the EGFP expression vector of pC-PgpdA-EGFP-TtrpC-neo. Then, the coding region of the target genes without stop codons was amplified using the primers listed in Table S2. After being digested with *Xba*Ⅰ and *EcoR*Ⅰ, the target genes were subcloned into the cloning sites of the EGFP expression vector between *PgpdA* and *egfp*. The localization expression vector of MrPigA was transformed into ∆*mrpigN* strains to obtain MrPigA-EGFP transformants. The localization expression vectors of MrPigC, MrPigN, MrPigE, and MrPigF were transformed into ∆*mrpigA*+*lig4*+*G*::*G* strains, respectively, to obtain MrPigC-EGFP, MrPigN-EGFP, MrPigE-EGFP, and MrPigF-EGFP transformants. All transformants were verified by PCR of the target genes. 

The transformants containing an EGFP label were observed under the EGFP channel. For the purpose of exploring more precise locations of MrPigA, the mycelia of the MrPigA-EGFP were dyed with Mito-Tracker Red CMXRos (Beyotime, Shanghai, China), a dye specifically designed for staining mitochondria. The fluorescence signal of the stained samples was detected using the EGFP and Mito Tracker Red (excitation wavelength: 561 nm, emission wavelength: 570–670 nm) channels. Thus, the precise location could be inferred based on the distribution and intensity of the EGFP signal and Mito-Tracker Red signal.

### 2.4. Study of the Role of MrPigF in MPs Biosynthesis

#### 2.4.1. Bioinformatics Analysis of MrPigF

MrPigF, the last synthetase in the MPs biosynthetic pathway, is responsible for catalyzing the synthesis of OMPs [5,20]. The full-length cDNA sequence of *mrpigF* was obtained by the rapid amplification of cDNA ends (RACE) technology using the primers in Table S3, as previously described [27]. The longest open reading frame (ORF) in the full-length cDNA sequence of the *mrpigF* gene was identified by the ORF finder (https://www.ncbi.nlm.nih.gov/orffinder, accessed on 8 June 2022), which was the coding DNA sequence (CDS) of the *mrpigF* gene, and the corresponding amino acid sequence was the amino acid sequence of the MrPigF protein. The SnapGene software version 6.02 was used to analyze the gene structure of the *mrpigF* gene, including the location of the CDS, introns, and the untranslated region (UTR). 

SignalP 6.0 was used to predict the signal peptide of MrPigF [31]. In addition, we collected all the published genome of the *Monascus* strains from NCBI database (https://www.ncbi.nlm.nih.gov/datasets/genome/?taxon=5097, accessed on 2 March 2023) to identify *mrpigF* gene sequences from the different species, and we then deduced the corresponding amino acid sequences. These amino acid sequences were compared by the Jalview software version 2.11 [32] and the Muscle program [33].

#### 2.4.2. Investigation of MrPigF Function in OMPs Biosynthesis

Based on the subcellular localization results of MrPigF, we prepared protoplasts of the MrPigF-EGFP strain using the method described by [34]. Then, the protoplasts were placed on a slide and observed under the EGFP channel. Moreover, a localization expression vector of MrPigF lacking the N-terminal signal peptide (MrPigF^∆SP^) was constructed as mentioned above. The localization expression vectors of MrPigF and MrPigF^∆SP^ were transformed into ∆*mrpigN* strains, respectively, to obtain the ∆*mrpigN*::MrPigF-EGFP and ∆*mrpigN*::MrPigF^∆SP^-EGFP transformants. Moreover, the resistant marker gene (*neo*) in the localization expression vectors of MrPigF and MrPigF^∆SP^ was replaced with the hygromycin-resistant gene (*hyg*). Those vectors were transformed into ∆*mrpigF* strains, respectively, to obtain the complementary transformants (∆*mrpigF*::MrPigF-EGFP strain and ∆*mrpigF*::MrPigF^∆SP^-EGFP strain). All the used primers are listed in Table S2. All transformants were verified by PCR of the target genes. The fluorescence signals were observed under the EGFP channel. 

### 2.5. Extraction and Detection of MPs

A total of 100 μL of a conidial suspension (10^5^ spores/mL) of *M. ruber* M7 and its transformants were inoculated on PDA media with sterilized cellophane and cultivated at 28 °C for 10 days, and the mycelia were then collected and lyophilized. A total of 20 mg of mycelia were fully mixed with 1.5 mL of extraction solution (methanol–water–formic acid ratio = 8:1.99:0.01, *v*/*v*/*v*). The ultrasonic extraction was carried out at 30 °C for 30 min. The supernatant containing MPs was collected after centrifuging at 12,000× *g* for 10 min. Then, the supernatant was filtered through a 0.22 μm filter membrane for chromatographic analysis. Four kinds of YMPs (monascin, ankaflavin, monasfluore A, monasfluore B) and two kinds of OMPs (rubropunctatin, monascorubrin) were separated and detected using high-performance liquid chromatography (HPLC, LC-20A, Shimadzu, Japan) with a C18 chromatography column (Inertsil ODS-3, 4.6 mm × 250 mm, 5 μm). The mobile phase included solvent A (acetonitrile) and solvent B (0.1% formic acid water). The flow rate and injection volume were 0.8 mL/min and 10 μL, respectively. The MPs were measured at 380 nm, 470 nm, and 520 nm under the following conditions: 0 min, 55% A and 45% B; 0–3 min, 65% A and 35% B; 3–30 min, 90% A and 10% B; and 30–35 min, 55% A and 45% B. The column temperature was maintained at 40 °C. 

### 2.6. RNA Extraction, cDNA Synthesis, and qRT-PCR

The mycelial samples were pre-frozen in a −80 °C freezer (Haier, Qingdao, China) and ground to a fine powder using liquid nitrogen; the total RNA was extracted with a TransZol Up Plus RNA Kit (TransGen, Beijing, China). Only RNA samples of a high purity (OD260/280 = 1.8∼2.2) and integrity (where two sharp bands of ribosomal RNAs were observed, and the intensity of the larger band was approximately twice that of the smaller band) were used for the reverse transcription experiments. cDNA was synthesized using a HiScript II 1st Strand cDNA Synthesis Kit (Vazyme, Nanjing, China) according to the instructions of the manufacturer. Reaction mixtures totaling 20.0 μL were used for the qRT-PCR, which contained 10.0 μL of 2 × AceQ qPCR SYBR Green Master Mix (Vazyme, Nanjing, China), 0.4 μL of each primer (10 μM each), 2.0 μL of cDNA sample, and 7.2 μL of ddH_2_O. qRT-PCR was performed using a qTOWER 2.2 real-time PCR system (Analytikjena, Jena, Thuringia, Germany) with a three-step application program (step 1, 95 °C for 5 min; step 2, 40 cycles of 94 °C for 10 s and 60 °C for 30 s; and step 3, 60 °C for 5 min). The relative expression levels for each target cDNA were calculated using the 2^−∆∆CT^ method [35]. The β-actin gene (GenBank accession no. AJ417880) served as a reference gene to normalize the values. All the used primers are listed in Table S1.

### 2.7. Statistical Analysis

Statistical data are expressed as means ± standard errors (SEs) from three replicates. Significance was assessed by one-way analysis of variance (ANOVA) using the GradPad Prism 8.0 software (Boston, MA, USA).

## 3. Results

### 3.1. MPs-Free Mutants Are Suitable for the Subcellular Localization Assay of M. ruber M7

**Figure 1:**
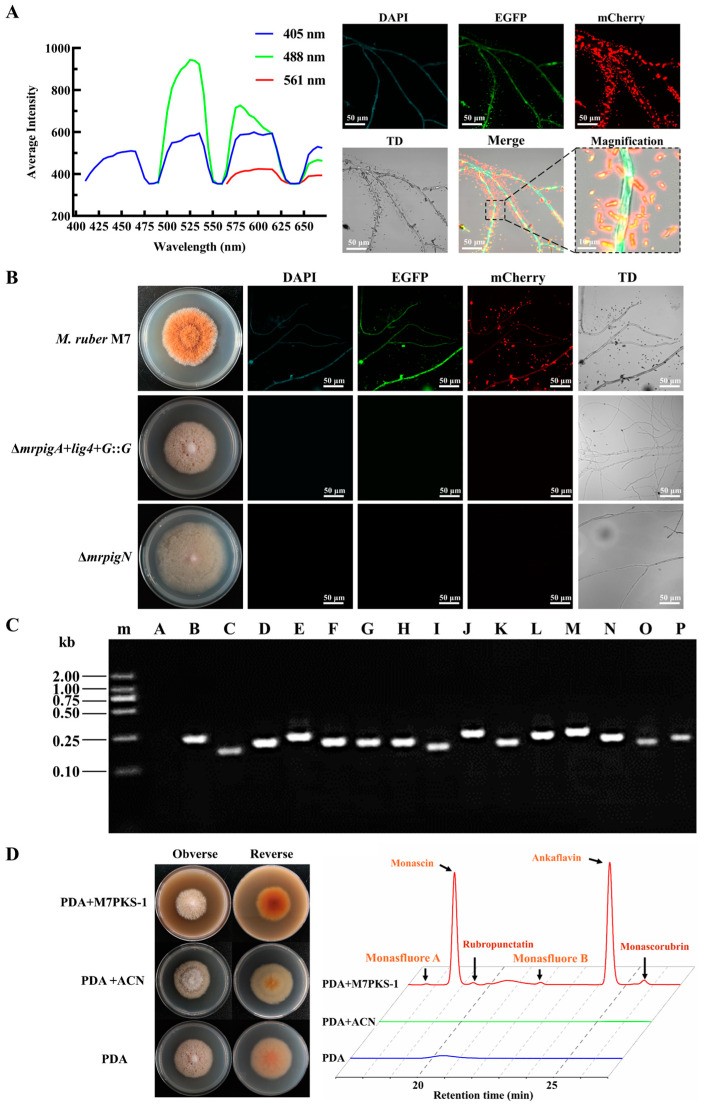
Analysis of spontaneous fluorescence of *M*. *ruber* M7 and characteristics of ∆*mrpigA*+*lig4*+*G*::*G* mutants. (**A**) Analysis of spontaneous fluorescence of *M*. *ruber* M7. Full-wavelength fluorescence scanning of *M. ruber* M7 mycelia was carried out using a confocal laser scanning microscope. Different levels of intrinsic fluorescence were observed in the *M. ruber* M7 mycelia when excited with wavelengths of 405 nm, 488 nm, and 561 nm. (**B**) Colony morphology and spontaneous fluorescence observation of M7, ∆*mrpigA*+*lig4*+*G*::*G,* and ∆*mrpigN* mutants under different channels. TD: transmitted light. (**C**) Verification of the gene transcription in MPs biosynthetic gene cluster of ∆*mrpigA*+*lig4*+*G*::*G* mutants. The cDNA from the ∆*mrpigA*+*lig4*+*G*::G mutants was used as a template to confirm the transcription of multiple genes within the MPs gene cluster utilizing the primers illustrated in Table S1. m: Trans 2K marker; A: *mrpigA*; B: *mrpigB*; C: *mrpigC*; D: *mrpigD*; E: *mrpigE*; F: *mrpigF*; G: *mrpigG*; H: *mrpigH*; I: *mrpigI*; J: *mrpigJ*; K: *mrpigK*; L: *mrpigL*; M: *mrpigM*; N: *mrpigN*; O: *mrpigO*; P: *mrpigP*. (**D**) M7PKS-1 feeding experiment. ∆*mrpigA*+*lig4*+*G*::*G* mutants were cultivated on PDA medium, PDA + ACN medium, and PDA + M7PKS-1 medium. ACN: acetonitrile. The content of MPs was analyzed using HPLC, with the chromatogram at a wavelength of 380 nm presented on the right.

The fluorescence spectrum scanning results showed that the hyphae of *M. ruber* M7 exhibited varying degrees of spontaneous fluorescence under the DAPI, EGFP, and mCherry channels (Figure 1A), suggesting that *M. ruber* M7 possesses spontaneous fluorescence even though it does not carry any fluorescent genes. Moreover, the magnified images showed the presence of some lamellar and needle-like crystals near the hyphae, which was consistent with the crystalline shapes of YMPs (monascin, ankaflavin) and OMPs (rubropunctatin, monascorubrin) [36]. These crystals exhibited significantly stronger spontaneous fluorescence compared to the hyphae. It was speculated that the MPs around the mycelia were responsible for the spontaneous fluorescence. Hence, we analyzed the fluorescence of two non-pigment-producing strains that were previously generated within our research group. One of them was the *mrpigA* deletion strain (∆*mrpigA+lig4+G*::*G*), which was engineered using a traceless and efficient gene modification system established in our previous work [4], whereas the other was the *mrpigN* deletion strain (∆*mrpigN*), which contains a *hyg* resistance gene [16]. As depicted in Figure 1B, both mutants exhibited white colonies and lacked fluorescence under fluorescent fields.

Furthermore, we conducted an analysis of the expression of MPs gene clusters in the ∆*mrpigA+lig4+G*::*G* strain. The results demonstrated that the remaining genes in the MPs biosynthesis gene cluster (*mrpigB*-*mrpigP*) showed detectable transcription levels in the *mrpigA* deletion strain (Figure 1C). Moreover, supplementation of the MPs intermediate M7PKS-1 resulted in the restoration of MPs production in the ∆*mrpigA*+*lig4*+*G*::*G* strain (Figure 1D). These findings suggest that the ∆*mrpigA*+*lig4*+*G*::*G* strain and ∆*mrpigN* were suitable for the subcellular localization experiments without interference from spontaneous fluorescence. Additionally, the disappearance of fluorescence in the absence of the MPs confirms that the spontaneous fluorescence of M7 was predominantly induced by the MPs, aligning with a previous study [37].

### 3.2. The Enzymes Involved in MPs Biosynthesis Are Compartmentalized in Various Subcellular Locations

**Figure 2:**
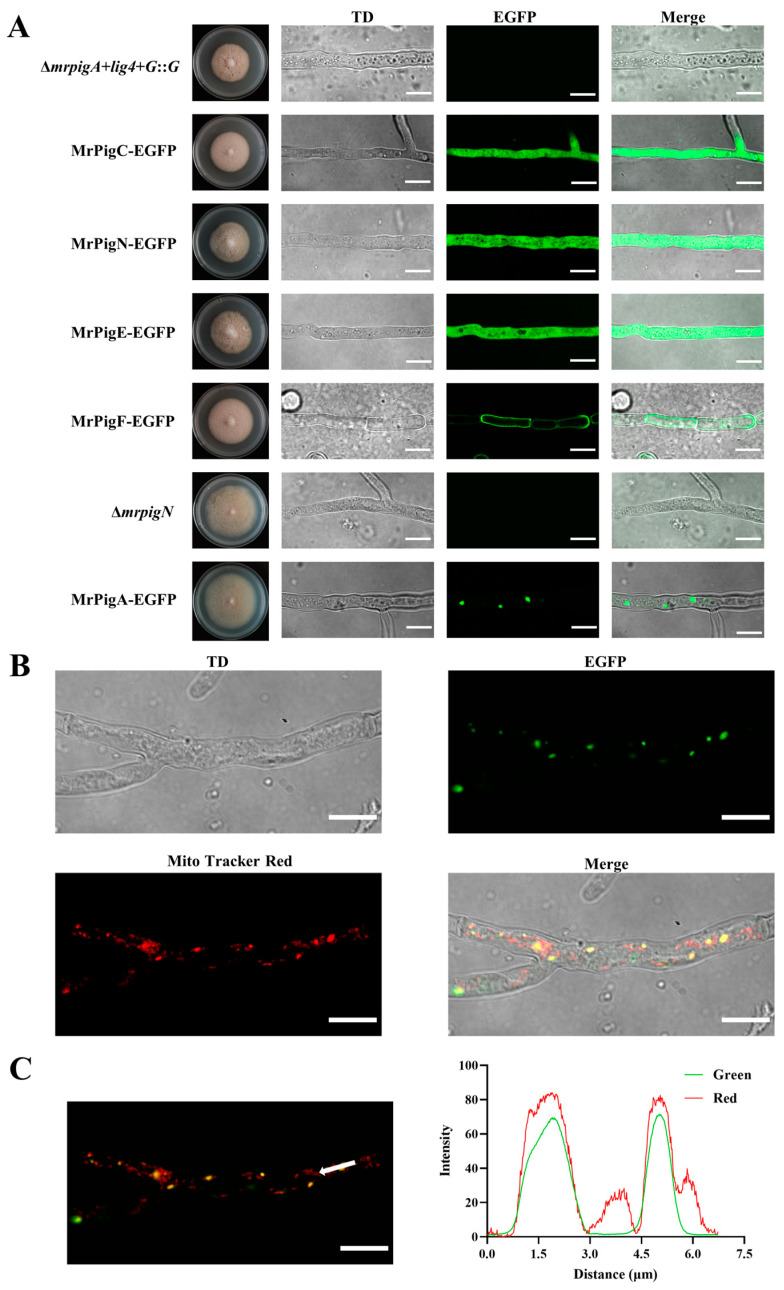
Subcellular locations of five key enzymes involved in the biosynthesis of MPs. (**A**) High-resolution confocal images for subcellular localizations of MrPigA, MrPigC, MrPigE, MrPigF, and MrPigN. The mutants were cultivated on PDA media at 28 °C for 7 d. TD: transmitted light. (**B**) Observations of TD, EGFP, and Mito-Tracker Red and merged image of the three channels in MrPigA-EGFP mutants are shown. TD: transmitted light. (**C**) Observations of merged EGFP and Mito-Tracker Red in MrPigA-EGFP mutants are shown; the white arrow indicates the position and direction of the fluorescence intensity analysis. Bars equal 10 μm.

To localize the biosynthesis of MPs within the *Monascus* cells, five crucial enzymes, MrPigA, MrPigC, MrPigE, MrPigF, and MrPigN, were tagged with an EGFP protein at their C-termini. The high-resolution confocal images clearly showed that these proteins were localized in different subcellular compartments (Figure 2A). Fluorescence signals were evenly distributed in the entire hyphae of the MrPigC-EGFP, MrPigN-EGFP, and MrPigE-EGFP mutants, indicating that MrPigC, MrPigN, and MrPigE were located in the cytosol. In addition, fluorescence signal resembling small dots appeared in the MrPigA-EGFP strain, revealing the potential existence of MrPigA in small vesicle structures. Fluorescence was exclusively detected at the periphery of the hyphae in the MrPigF-EGFP strain, indicating the localization of MrPigF at the hyphal edge. 

To further determine the specific localization of MrPigA, the mycelia of the MrPigA-EGFP strain were stained using a mitochondria-specific dye (Mito-Tracker Red CMXRos). The mycelia were observed under the EGFP and Mito-Tracker Red channels. The merged picture showed that the distribution of the green fluorescent signal closely overlapped with the red fluorescent signal (Figure 2B). The intensities of green fluorescence and red fluorescence were almost the same (Figure 2C). The above results provide persuasive evidence that MrPigA is localized in mitochondria. 

### 3.3. The Correct Localization of MrPigF to the Cell Wall Is Crucial for the Synthesis of OMPs

#### 3.3.1. Sequence Analysis of mrpigF Gene

**Figure 3:**
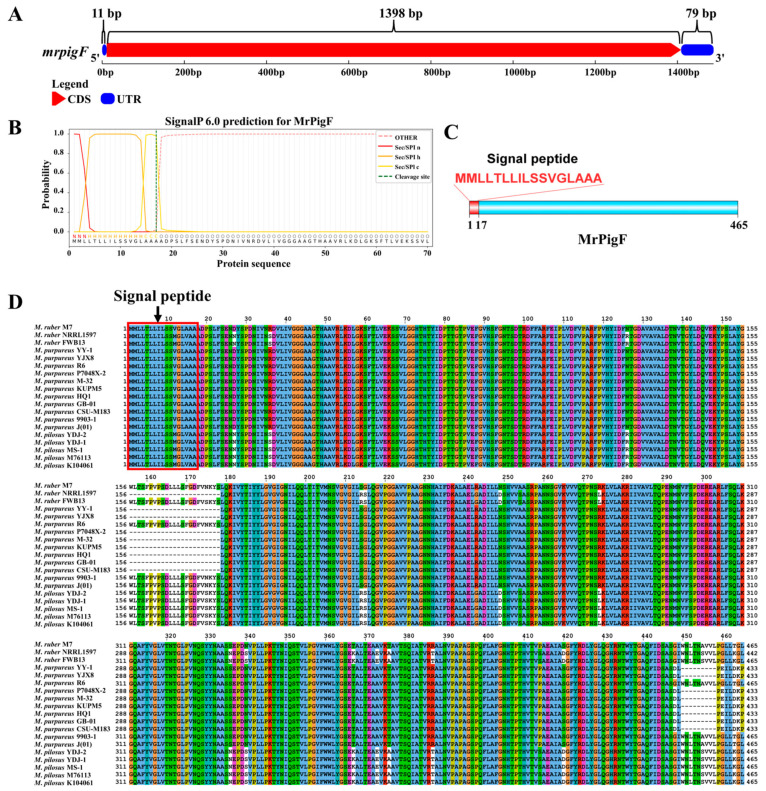
Bioinformatics analysis of MrPigF. (**A**) The gene structure diagram of *mrpigF*. The full-length cDNA sequence of *mrpigF* was determined through RACE, and it is presented in Figure S2. (**B**) Prediction of signal peptide sequence in MrPigF using SignalP 6.0. (**C**) The protein structure diagram of MrPigF. (**D**) Multiple sequence alignment of MrPigF protein of nineteen *Monascus* strains.

Given the specialized subcellular localization and functional role of MrPigF in synthesizing OMPs, we conducted a comprehensive investigation into MrPigF. The full length of the *mrpigF* gene was 1488 bp, consisting of only one exon and no introns. The lengths of the 5’UTR, 3’UTR, and CDS regions were 11 bp, 79 bp, and 1398 bp, respectively (Figure 3A). The amino acid sequence of MrPigF was analyzed using SignalP 6.0, and the results are shown in Figure 3B,C. The *mrpigF* gene encoded 465 amino acids. The N-terminus of MrPigF was a classical signal peptide (SP) consisting of 17 amino acids with the sequence “MMLLLTLLILSSVGLAAA”, followed by a splice site, indicating that MrPigF was a typical secreted protein. In addition, we collected all the published genomes of *Monascus* spp. from NCBI, including 2 *M. ruber* strains, 11 *M. purpureus* strains, and 5 *M. pilosus* strains. The amino acid sequences of MrPigF in these strains and *M. ruber* M7 were subjected to multiple sequence comparison. As shown in Figure 3D, the front end of the MrPigF protein in those *Monascus* strains possessed a highly conserved signal peptide sequence. 

#### 3.3.2. Protein Structure Influences the Subcellular Localization of MrPigF

**Figure 4:**
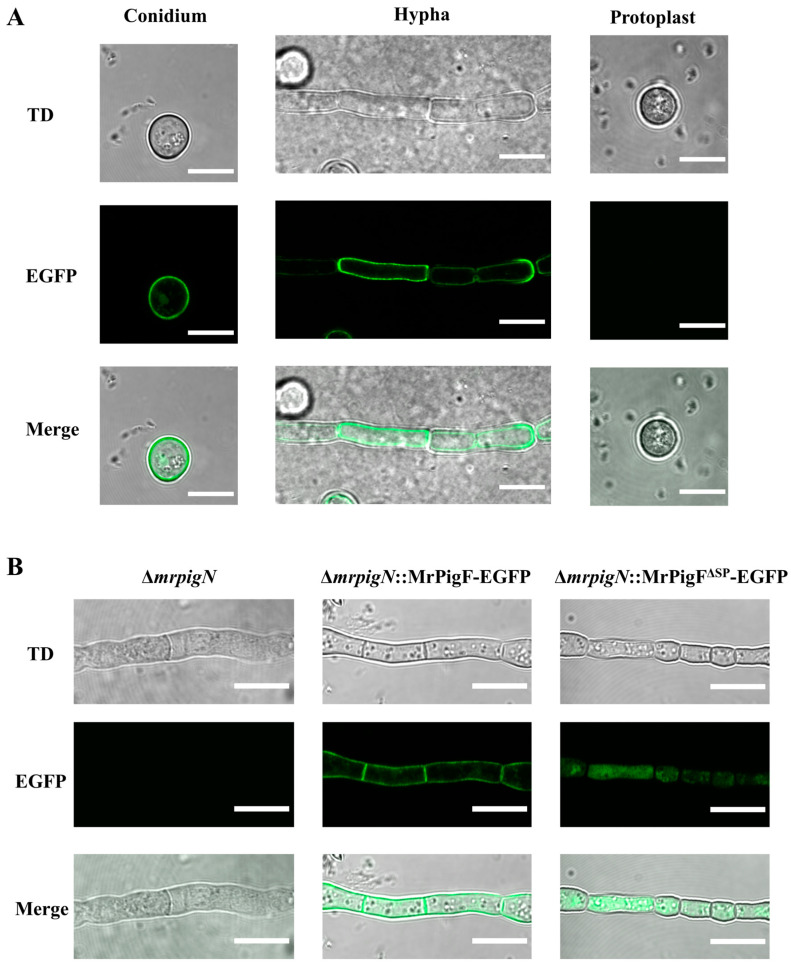
Subcellular localization analysis of MrPigF. (**A**) Subcellular localization analysis of MrPigF. (**B**) Subcellular localization analysis of MrPigF^∆SP^. TD: transmitted light. Bars equal 10 μm.

The subcellular localization of MrPigF was further determined by removing the cell wall of the MrPigF-EGFP strain. As shown in Figure 4A, the fluorescence signals were emitted from the edges of the conidia and hyphae, but no obvious fluorescence signals were observed in the protoplasts without a cell wall. Therefore, our results confirmed that MrPigF was localized in the cell wall of M7. 

To investigate whether MrPigF was localized to the cell wall through a signal peptide, we constructed the ∆*mrpigN*::MrPigF^∆SP^-EGFP (MrPigF without SP) and ∆*mrpigN*::MrPigF-EGFP mutants, since ∆*mrpigN* had a higher conversion efficiency as the starting strain. As shown in Figure 4B, although the fluorescence intensity of the two mutants was almost the same, the location of the fluorescence was significantly different. The fluorescence was emitted from the cell wall in the ∆*mrpigN*::MrPigF-EGFP mutants, which was consistent with the above subcellular location analysis of MrPigF. The absence of the N-terminal SP resulted in the incorrect localization of MrPigF^∆SP^ to the cytoplasm. These results demonstrate that the N-terminal SP is significant for directing MrPigF cell wall localization.

#### 3.3.3. MrPigF Mediates the Synthesis of OMPs on the Cell Wall

**Figure 5:**
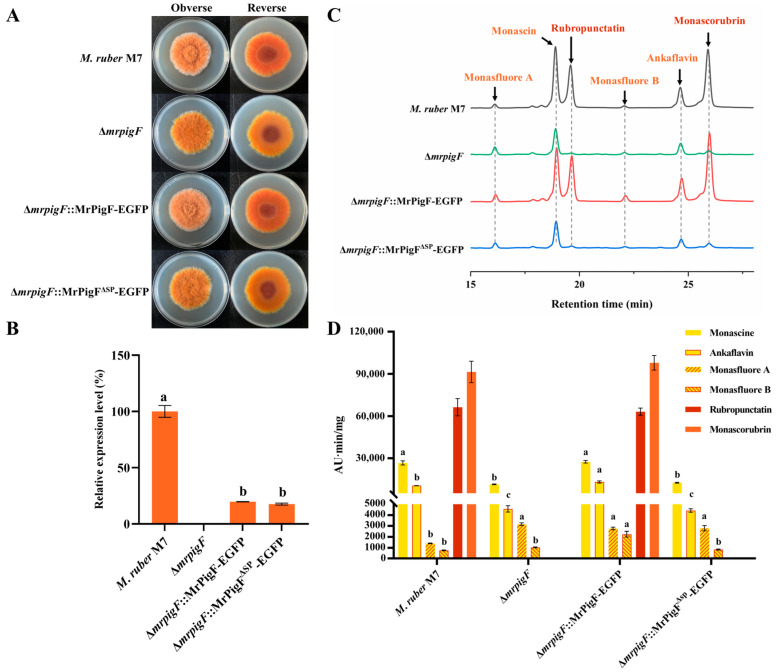
Effects of subcellular localization of MrPigF on MPs production. (**A**) Colony morphology on PDA media. (**B**) The expression level of *mrpigF* in the indicated strains was determined by qRT-PCR. Error bars represent the standard error of three biological replicates; different letters in the graph indicate statistical significance between strains (one-way ANOVA) (*p* < 0.05). (**C**) MPs species analysis of different strains cultivated on PDA media for 7 days. (**D**) MPs from different strains were analyzed and quantified using HPLC. Error bars represent the standard error of three biological replicates. Different letters in the graph indicate statistical significance of each MPs among different mutants. In alphabetical order, a preceding position signifies a greater magnitude of statistical variance (one-way ANOVA) (*p* < 0.05).

To investigate the role of MrPigF localization in the synthesis of MPs, the ∆*mrpigF*::MrPigF-EGFP strain and ∆*mrpigF*::MrPigF^∆SP^-EGFP strain were constructed and analyzed. The colony color of the ∆*mrpigF*::MrPigF-EGFP strain exhibited an orange-yellow color, which was consistent with the color of the wild-type strain M7. The colony color of the ∆*mrpigF*::MrPigF^∆SP^-EGFP strain showed a bright yellow color, which was the same as the color of the ∆*mpigF* mutants (Figure 5A). The results of the qRT-PCR analysis confirmed that the *mrpigF* gene was knocked out in the ∆*mrpigF* strain, while the expression of *mrpigF* was redetected in the ∆*mrpigF*::MrPigF-EGFP and ∆*mrpigF*::MrPigF^∆SP^-EGFP strains, suggesting that MrPigF and MrPigF^∆SP^ were successfully complemented back into the ∆*mrpigF* strain, respectively (Figure 5B). As seen in Figure 5C,D, four YMPs (monascin, ankaflavin, monasfluore A, and monasfluore B) and two OMPs (rubropunctatin and monascorubrin) were detected in the M7 mycelia. Only four YMPs were detected in the *mrpigF*-deleted strain, which lost the ability to produce OMPs. The results were consistent with a previous report [5]. The ∆*mrpigF*::MrPigF-EGFP strain regained the ability to produce OMPs and had a similar yield of two OMPs compared to M7. However, lacking the N-terminal SP in the ∆*mrpigF*::MrPigF^∆SP^-EGFP strain resulted in the absence of OMPs, indicating that the N-terminal SP played a vital role in OMPs biosynthesis. Our study confirmed that MrPigF was localized to the cell wall, while MrPigF^∆SP^ was distributed in the cytoplasm. Therefore, the correct localization of MrPigF to the cell wall is crucial for the synthesis of OMPs in *M. ruber*. 

Additionally, the contents of four YMPs in the three mutants ranged to varying degrees. Knocking out *mrpigF* resulted in a significant decrease in monascin and ankaflavin but an increase in monasfluore A and monasfluore B. In the ∆*mrpigF*::MrPigF-EGFP mutants, the biosynthesis of YMPs, particularly ankaflavin, monasfluore A, and monasfluore B, was enhanced compared to M7. However, the absence of the N-terminal SP in the ∆*mrpigF*::MrPigF^∆SP^-EGFP strain led to a significant decrease in monascin and ankaflavin but a remarkable increase in monasfluore A. OMPs (rubropunctatin, monascorubrin) can be converted into YMPs (monascin, ankaflavin) in *Monascus* [19]. Thus, the two YMPs, monascin and ankaflavin, were significantly decreased in the ∆*mrpigF* and ∆*mrpigF*::MrPigF^∆SP^-EGFP strains, probably because of the absence of OMPs. Moreover, the precursors that should be used for the biosynthesis of OMPs were diverted to the biosynthesis of monasfluore A and monasfluore B, as shown in Figure S1, leading to an increase in the content of monasfluore A and monasfluore B.

## 4. Discussion

In *M*.*ruber* M7, the biosynthetic gene cluster of MPs contains 16 genes, encoding 15 proteins (MrPigJ and MrPigK jointly encode MrPigJ-K) [5]. Both MrPigB [38] and MrPigI [37] are transcription factors in the gene cluster and are theoretically located in the cell nucleus. According to previous reports, MrPigL [39] and MrPigG [40] play a small role in the biosynthesis of MPs. Therefore, our research focused on 11 proteins, including MrPigA, MrPigC, MrPigD, MrPigE, MrPigF, MrPigH, MrPigJ-K, MrPigM, MrPigN, MrPigO, and MrPigP. In this study, experiments revealed that the early synthesis enzyme of MPs, MrPigA, is located in the mitochondria, while the mid-stage synthesis enzymes MrPigC, MrPigN, and MrPigE are located in the cytoplasm, and the late-stage synthesis enzyme MrPigF is located in the cell wall. To enhance the comprehensiveness of the entire compartment synthesis pathway, we predicted the subcellular localization of the remaining six enzymes using WoLF PSORT II [41]. The localization results of the 11 MPs synthesis proteins obtained from the experiments and bioinformatics predictions are shown in Table 2.

It is well known that the SMs of many filamentous fungi are their “armor” and “weapons” against environmental and biological competition [23,42]. OMPs possess powerful antimicrobial activity, and the derivatives of OMPs exhibit even stronger antimicrobial activity after reacting with amino acids [11]. Therefore, OMPs serve as the “weapons” of *Monascus* spp. to compete against other organisms. However, the antimicrobial activity can also act on themselves [10]. Duan et al. discovered that MrPigE was responsible for converting OMPs into YMPs using in vitro and in vivo assays [19]. Interestingly, our results confirmed that MrPigE was scattered in the cytoplasm, and OMPs were only synthesized in the cell wall. Consequently, we proposed that when an excessive accumulation of OMPs on the cell wall may enter to the cell, MrPigE converts them into YMPs without antimicrobial activity to protect the *Monascus* itself. Furthermore, the N-terminal SP is significant for directing MrPigF’s cell wall localization. Our study showed that the absence of the N-terminal SP resulted in the incorrect localization of MrPigF^∆SP^ to the cytoplasm instead of directing it to the cell wall, resulting in the loss of the ability to produce OMPs. This phenomenon might be attributed to the enzymatic action of MrPigE, which catalyzes the conversion of intracellular OMPs into YMPs. Similarly, Lim et al. reported that FmqD was characterized as a secreted protein because a conserved SP sequence was located at the N-terminus [26]. The FmqD incorrectly reached the cytoplasm when the SP sequence was removed, leading to an increase in the production of FqA and a decrease in the yield of FqC, indicating that the correct localization of FmqD is critical for FqC synthesis. The right subcellular localization of MrPigF is significant for MPs biosynthesis.

In earlier research, it was demonstrated that MrPigP belonged to the major facilitator superfamily (MFS), but its function in MPs synthesis had not been clearly defined [5]. We found that MrPigP had 14 transmembrane domains, suggesting that it may be located on the cell membrane. The production of YMPs significantly increased, while that of OMPs remarkably decreased in the ∆*mrpigP* mutants [39]. MrPigF and MrPigH were reported to be responsible for the synthesis of OMPs (rubropunctatin, monascorubrin) and YMPs (monascin, ankaflavin), respectively, using the same MPs precursors [5,20]. According to our results of localization, MrPigF and MrPigH were located at the cell wall (extracellular) and in the cytoplasm (intracellular). Considering the function of the MFS, it is speculated that the deletion of *mrpigP* reduced the efficiency of transporting MPs precursors to the extracellular environment, which resulted in a lower yield of OMPs in the ∆*mrpigP* mutants. Hence, MrPigP might play a role in facilitating the extracellular transportation of MPs precursors, which is not crucial but could improve the efficacy of conveying these precursors to the external milieu.

**Figure 6:**
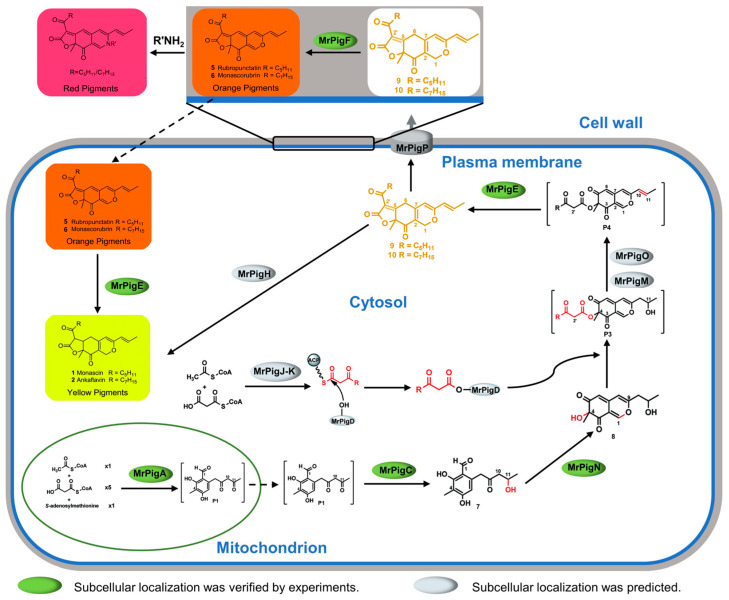
Schematic compartmentalized MPs biosynthesis.

Based on the above results and the MPs synthesis pathway, the compartmentalized synthesis pathway of MPs is proposed. Overall, the subcellular locations of the key enzymes involved in MPs biosynthesis are exhibited in Figure 6. The non-reducing polyketide synthase MrPigA catalyzed the intermediate P1 in the mitochondria using one unit of acetyl coenzyme A (CoA), five units of acetyl CoA, and one unit of S-adenosylmethionine as starting substances. The intermediate P1 entered the cytoplasm and was subsequently catalyzed by the keto-reductase MrPigC and monooxygenase MrPigN in sequence to synthesize intermediate 8. Then, intermediate 8 was catalyzed by the acyltransferase MrPigD to form the intermediate P3 after adding a fatty acid side chain, which was synthesized by the fatty acid synthase MrPigJ-K in the cytoplasm. Subsequently, the intermediate P3 was transformed into the intermediate P4 under the catalysis of oxidoreductase MrPigM and MrPigO. Then, the oxidoreductase MrPigE catalyzed intermediate P5 to generate MPs precursors 9 and 10. The MPs precursors were catalyzed by the enoyl reductase MrPigH in the cytoplasm to synthesize YMPs (1: monascin, 2: ankaflavin). Furthermore, the MPs precursors were transported to the extracellular environment with the assistance of MrPigP and then catalyzed by the FAD-dependent oxidoreductase MrPigF on the cell wall to produce antimicrobial OMPs (5: rubropunctatin, 6: monascorubrin). If OMPs enter the intracellular environment, they could be catalyzed by MrPigE into YMPs. In addition, extracellular orange MPs could react with amine substances to produce a variety of red MPs.

Compartmentalization engineering is a metabolic engineering strategy targeting metabolic pathways within specific cellular compartments to optimize metabolic pathways and enhance the production of specific compounds [43,44,45]. In recent years, compartmentalization engineering has been successfully applied to the synthesis of secondary metabolites of various fungi. For example, Zhu et al. proposed a combinatorial strategy of cytoplasmic and mitochondrial engineering to alleviate the metabolic burden of mevalonate biosynthesis in yeasts, and the highest production of squalene titer approached 21.2 g/L [46]. Blumhoff et al. reported that the itaconic acid biosynthesis pathway was most efficient in mitochondria, and the yield of itaconic acid produced by *Aspergillus* species in the mitochondria was 2-fold higher than that in the cytosol [47]. The production of *Aspergillus nidulans* penicillin was significantly improved by targeting AcvA from the cytosol to peroxisomes [48]. Based on the compartmentalized biosynthesis of MPs, the compartmentalization engineering strategy has great potential to be applied in the production of single and pure MPs. YMPs (monascin, ankaflavin) have been reported to be no less active in lowering blood lipids than monacolin K and have fewer side effects, thus possessing promising commercial value [9]. Single and high-purity YMPs can be efficiently synthesized by knocking out the *mrpigF* gene and targeting MrPigH from the cytosol to the cell wall.

In summary, the subcellular localization of five key MPs synthases was studied using the fluorescent protein fusion expression method, respectively. Furthermore, our investigation confirmed that the subcellular localization of MrPigF was influenced by protein structures. The correct localization of MrPigF to the cell wall was crucial for the synthesis of OMPs in *M. ruber*. Additionally, we also combined bioinformatics analysis to discuss the compartmentalized synthetic pathway of MPs. These findings offer new insights for a comprehensive understanding of the compartmentalization biosynthesis of MPs, and they provide the theoretical foundation for producing pure and high-quality MPs.

## Figures and Tables

**Table 1:** *M. ruber* M7 and the relative strains used in this study.

Strain	Genotype	Description	Source
*M*. *ruber* M7	Wild type		[28]
∆*mrpigN*	M7-∆*mrpigN*::*hyg*	*mrpigN* deletion strain	[16]
∆*mrpigE*	M7-∆*mrpigE*::*hyg*	*mrpigE* deletion strain	[18]
∆*mrpigF*	M7-∆*mrpigF*::*neo*	*mrpigF* deletion strain	[5]
∆*mrpigA*+*lig4*+*G*::*G*	M7-∆*mrpigA*∆*mrlig4*∆*mrpyrG*::*mrpyrG*	*mrpigA* and *mrlig4* deletion strain	Our lab
MrPigC-E*G*FP	M7-∆*mrpigA*∆*mrlig4*∆*mrpyrG*::*mrpyrG*::*PgpdA*-*mrpigC*-*egfp*::*neo*	Expressing *egfp*-tagged *mrpigC* gene in ∆*mrpigA*+*lig4*+*G*::*G*	This study
MrPigN-E*G*FP	M7-∆*mrpigA*∆*mrlig4*∆*mrpyrG*::*mrpyrG*::*PgpdA*-*mrpigN*-*egfp*::*neo*	Expressing *egfp*-tagged *mrpigN* gene in ∆*mrpigA*+*lig4*+*G*::*G*	This study
MrPigE-E*G*FP	M7-∆*mrpigA*∆*mrlig4*∆*mrpyrG*::*mrpyrG*::*PgpdA*-*mrpigE*-*egfp*::*neo*	Expressing *egfp*-tagged *mrpigE* gene in ∆*mrpigA*+*lig4*+*G*::*G*	This study
MrPigF-E*G*FP	M7-∆*mrpigA*∆*mrlig4*∆*mrpyrG*::*mrpyrG*::*PgpdA*-*mrpigF*-*egfp*::*neo*	Expressing *egfp*-tagged *mrpigF* gene in ∆*mrpigA*+*lig4*+*G*::*G*	This study
MrPigA-E*G*FP	M7-∆*mrpigN*::*hyg*::*PgpdA*-*mrpigA*-*egfp*::*neo*	Expressing *egfp*-tagged *mrpigA* gene in ∆*mrpigN*	This study
∆*mrpigN*::MrPigF-E*G*FP	M7-∆*mrpigN*::*hyg*::*PgpdA*-*mrpigF*-*egfp*::*neo*	Expressing *egfp*-tagged *mrpigF* gene in ∆*mrpigN*	This study
∆*mrpigN*::MrPigF^∆SP^-E*G*FP	M7-∆*mrpigN*::*hyg*::*PgpdA*-*mrpigF*^∆*SP*^-*egfp*::*neo*	Expressing *egfp*-tagged *mrpigF*^∆*SP*^ gene in ∆*mrpigN*	This study
∆*mrpigF*::MrPigF-E*G*FP	M7-∆*mrpigF*::*neo*::*PgpdA*-*mrpigF*-*egfp*::*hyg*	Expressing *egfp*-tagged *mrpigF* gene in ∆*mrpigF*	This study
∆*mrpigF*::MrPigF^∆SP^-E*G*FP	M7-∆*mrpigF*::*neo*::*PgpdA*-*mrpigF*^∆*SP*^-*egfp*::*hyg*	Expressing *egfp*-tagged *mrpigF*^∆*SP*^ gene in ∆*mrpigF*	This study

Note: *mrpigF*^∆*SP*^ gene refers to *mrpigF* without the N-terminal signal peptide coding sequence.

**Table 2:** Subcellular localization of MPs biosynthetic proteins.

Protein	Function	Localization	Method
MrPigA	Nonreducing polyketone synthase	Mitochondria	Experiment
MrPigC	C-11-ketoreductase	Cytosol	Experiment
MrPigD	4-O-acyltransferase	Cytosol	Prediction
MrPigE	NAD(P)H-dependent oxidoreductase	Cytosol	Experiment
MrPigF	FAD-dependent oxidoreductase	Cell Wall	Experiment
MrPigH	Enoyl reductase	Cytosol	Prediction
MrPigJ-K	Fatty acid synthetase	Cytosol	Prediction
MrPigM	O-acetyltransferase	Cytosol	Prediction
MrPigN	FAD-dependent monooxygenase	Cytosol	Experiment
MrPigO	Deacetylase	Cytosol	Prediction
MrPigP	Major facilitator superfamily	Cell membrane	Prediction

## Data Availability

The original contributions presented in the study are included in the article/Supplementary Materials, further inquiries can be directed to the corresponding authors.

## Supplementary Materials

The following supporting information can be downloaded at: https://www.mdpi.com/article/10.3390/jof10060375/s1, Table S1 Primers for transcription analysis. Table S2 Primers for the construction of EGFP-labeled mutants. Table S3 Primers for amplification of *mpigF* full-length cDNA sequence. Figure S1 MPs biosynthetic gene cluster and its biosynthetic pathway. (A) The biosynthetic gene cluster of MPs. (B) The biosynthetic pathway of MPs. P1–P6 in square brackets are proposed intermediates that may be too reactive to isolate, or not fully characterized. Figure S2 *mrpigF* gene full-length cDNA sequence.
