# Supplementary material for: Exploring the Subcellular Localization of Monascus Pigments Biosynthases: Preliminary Unraveling of the Compartmentalization Mechanism
Source: J Fungi (Basel). 2024 May 24;10(6):375. doi: 10.3390/jof10060375 (PMC11205011; doi:10.3390/jof10060375)
Supplement: Supplementary file 1 [file jof-10-00375-s001.zip › jof-3006497-supplementary.pdf]

Supplementary material

Supplementary materials legends

Table S1 Primers for transcription analysis.

Table S2 Primers for the construction of EGFP-labeled mutants.

Table S3 Primers for amplification of *mpigF* full-length cDNA sequence.

Figure S1 MPs biosynthetic gene cluster and its biosynthetic pathway. (A) The biosynthetic gene cluster of MPs. (B) The biosynthetic pathway of MPs. P1–P6 in square brackets are proposed intermediates that may be too reactive to isolate, or not fully characterized.

Figure S2 *mrpigF* gene full-length cDNA sequence.

Table S1 Primers for transcription analysis.

Primers	Sequences (5'→3')	Function
mrpigA-F(q)	CTCGAGGAATTGAGCGTTGG	Amplification of *mrpigA*, 186 bp
mrpigA-R(q)	CAGGAAGACTCAATTCGCCG	
mrpigB-F(q)	CAGAAACCATCACGCAGGAG	Amplification of *mrpigB*, 246 bp
mrpigB-R(q)	AAAGAAGCAGCGGGTCTACT	
mrpigC-F(q)	CCTACCCAGCAATCGATCCT	Amplification of *mrpigC*, 169 bp
mrpigC-R(q)	ACGTCCTTTGCTAGCTCTGT	
mrpigD-F(q)	GTACGCGGGGAAGTTCAATC	Amplification of *mrpigD*, 212 bp
mrpigD-R(q)	CCCCAATATCCTCCCTCGTC	
mrpigE-F(q)	CTGTACAACGTCCTGCATCG	Amplification of *mrpigE*, 247 bp
mrpigE-R(q)	TCTCCCGAATCGTATCCAGC	
mrpigF-F(q)	CTTCGGCAACACCTCGGA	Amplification of *mrpigF*, 201 bp
mrpigF-R(q)	GAAGTTAACCACCCGTAGGC	
mrpigG-F(q)	TACAAGGAGTTCGGGCCATT	Amplification of *mrpigG*, 199 bp
mrpigG-R(q)	GCAGGCTAGCACACATCTTC	
mrpigH-F(q)	TCGTCTCGTGGATCATCTCG	Amplification of *mrpigH*, 197 bp
mrpigH-R(q)	GATGCTCTCCAATCCCTTGC	
mrpigI-F(q)	CATCTTGGACGGGATTGCAG	Amplification of *mrpigI*, 162 bp
mrpigI-R(q)	ATCTCGTCCTTGCTCACACA	
mrpigJ-F(q)	CGTTTCGGCTGATCATTCGT	Amplification of *mrpigJ*, 236 bp
mrpigJ-R(q)	CGATCCCGCTGAAGAACTTG	
mrpigK-F(q)	CAATCGGACGGGAAATGACC	Amplification of *mrpigK*, 176 bp
mrpigK-R(q)	CTTTGAGTCTCATCGCCAGC	
mrpigL-F(q)	TCAGGGATTGTGGGATTGCT	Amplification of *mrpigL*, 220 bp
mrpigL-R(q)	CTTGCATCGCCTTGTCAACT	
mrpigM-F(q)	GTGACTTTGAACAGCCTGGG	Amplification of *mrpigM*, 241 bp
mrpigM-R(q)	CGCTCAATTCCTTCTCCAGC	
mrpigN-F(q)	CGATGCAATGGGGAGAGAGA	Amplification of *mrpigN*, 200 bp
mrpigN-R(q)	CGAATCCAGAGAAGGCTTGC	
mrpigO-F(q)	AACTGCTCTTCGAGACGGAT	Amplification of *mrpigO*, 168 bp
mrpigO-R(q)	CGAACTCCAGCAGCAACTTC	
mrpigP-F(q)	CTATTTGGTGCGGACGAGTG	Amplification of *mrpigP*, 186 bp
mrpigP-R(q)	TCCAACACCTCTTCGATGCT	
β-actin-F(q)	TCTGGCACCACACATTCTACAA	Amplification of *β-actin*, 120 bp
β-actin-R(q)	CGAAGACGATCTGGGTCATCT	

Table S2 Primers for the construction of EGFP-labeled mutants.

Name	Sequences (5'→3')	Function
gpdaF(kpn)	CGGGCAGGACCGGACGGGGCGGTACGCCCCGAAGTGGAAAGGC	Amplification of *PgpdA*, 698 bp
gpdaR(egfp)	AGCTCCCACTGGATCCACTCGAGCCGAATTCCTGCAGCCCGGG	
egfpF(gpda)	GGCTCGAGTGGATCCAGTGGGAGCTCTGGGAGTAGTATGGTGAGCAAGGGCGAGG	Amplification of *egfp*, 781 bp
egfpR(TrpC)	CTAGAAGGCACTCTTTGCTGCTTGGTTACTTGTACAGCTCGTCCATGC	
TtrpCF(egfp)	CCAAGCAGCAAAGAGTGCCTTCTAG	Amplification of *TtrpC*, 624 bp
TtrpCR(neo)	GGTTACGGTTCGATGGGGTTGAGTTGGCTAGAAAGAAGGATTACCTC	
neoF(TrpC)	CCAACTCAACCCCATCGAACCGTAACC	Amplification of *neo*, 1246 bp
neoR(Pst)	CCAGTGCCAAGCTTGCATGCCTGCAATCATCATGCAACATGCATGTA	
pigA-1F	CCAAGAACCTTTATTTGCGGCCGCTTATGGTCAGTCGGATATTGCGC	For segmental amplification of *mrpigA* gene fragments with lengths of 2800 bp, 2660 bp and 2714 bp, respectively
pigA-1R	AAGCCTACAAATGTGGAGATCTGACCA	
pigA-2F	TGGTCAGATCTCCACATTTGTAGGCTT	
pigA-2R	AAGAATCAACCCCAAGATCACGGAGG	
pigA-3F	CCTCCGTGATCTTGGGGTTGATTCTT	
pigA-3R	CACTGGATCCACTCGAGCCGAATTCGTGCAGGAAACCCATCTCCTTCC	
pigCF	AACCTTTATTTGCGGCCGCTCTAGAATGCCTCCTCCTAGGGGTAC	Amplification of *mrpigC*, 959 bp
pigCR	CACTGGATCCACTCGAGCCGAATTCGTAGATAAATTTCACTTTCAACTTGTCTCC	
pigNF	AACCTTTATTTGCGGCCGCTCTAGAATGCCCGGAGTCACGAAAG	Amplification of *mrpigN*, 1358 bp
pigNR	CACTGGATCCACTCGAGCCGAATTCTTCCAGTCTACGTCTATAGTCTTCAGCC	
pigEF	AACCTTTATTTGCGGCCGCTCTAGAATGGGAAGCATTTCGCCCAA	Amplification of *mrpigE*, 1076 bp
pigER	CACTGGATCCACTCGAGCCGAATTCGTGATAGTAAGGCCCCACGACTG	
pigFF	AGAACCTTTATTTGCGGCCGCTCTAGAATGATGCTTCTCACGCTTCTCAT	Amplification of *mrpigF*, 1447 bp
pigFR	CACTGGATCCACTCGAGCCGAATTCAAGACCCGTCAACAGCCC	
pigF$^{\Delta SP}$F	AACCTTTATTTGCGGCCGCTCTAGAATGGCGGACCCATCTCTATTCAG	Amplification of *mrpigF*$^{\Delta SP}$, 1397 bp

gpdaF(kpn)	CGGGCAGGACCGGACGGGGCGGTACGCCCCGAAGTGGAAAGGC	Amplification of MrPigF-EGFP/MrPigF$^{\Delta SP}$-EGFP expression cassette, 3405 bp/3357 bp
TtrpCR(hyg)	ATCTTCTGTCGACCTAGAAAGAAGGATTACCTCTAAACAAGTGTACCT	
hygF	CTTCTTTCTAGGTCGACAGAAGATGATATTGAAGGAG	Amplification of hygromycin phosphotransferase gene *hyg*, 2169 bp
hygR	TAAAACGACGGCCAGTGCCACTAGAAAGAAGGATTACCTCTAAACAAGTGT	
gpdaF	GCCCCGAAGTGGAAAGGC	For the verification of vector construction and the screening and verification of mutant strains
egfpR	TTACTTGTACAGCTCGTCCATGC	
neoF	CCAACTCAACCCCATCGAACCGTAACC	
PtrpCR	ATCGATGCTTGGGTAGAATA	

Table S3 Primers for amplification of *mpigF* full-length cDNA sequence.

Primers	Sequences (5'→3')
3'OligodT	GCTGTCAACGATACGCTACGTAACGGCATGACAGTG(T)$_{18}$
3'adapter	GATACGCTACGTAACGGCATGACAG
5'adapter	GACTCGAGTCGACATCGA(C)$_{17}$
pigF-up	GCGGACCCATCTCTATTCAGC
pigF-do	AAGACCCGTCAACAGCCC
F-3'GSP1	TCACGTCTCAGATCGCAACGG
F-3'GSP2	ATCTGTACGGGCTTCAGGGTT
F-5'GSP1	TCGCCAAAGGACAGGAGCAGA
F-5'GSP2	AAAGAAGTCCCTGGTGTCCGAG

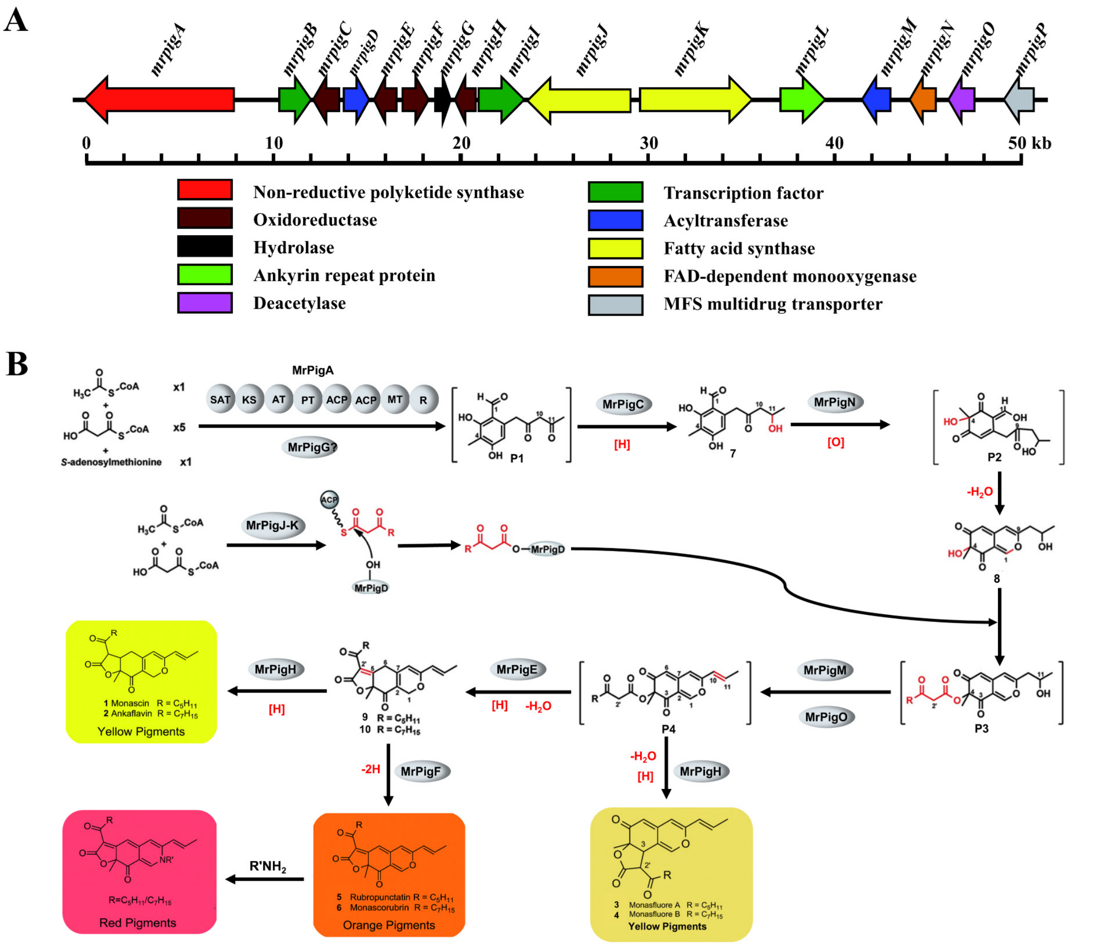

Figure S1 MPs biosynthetic gene cluster and its biosynthetic pathway. (A) The biosynthetic gene cluster of MPs. (B) The biosynthetic pathway of MPs. P1–P6 in square brackets are proposed intermediates that may be too reactive to isolate, or not fully characterized.

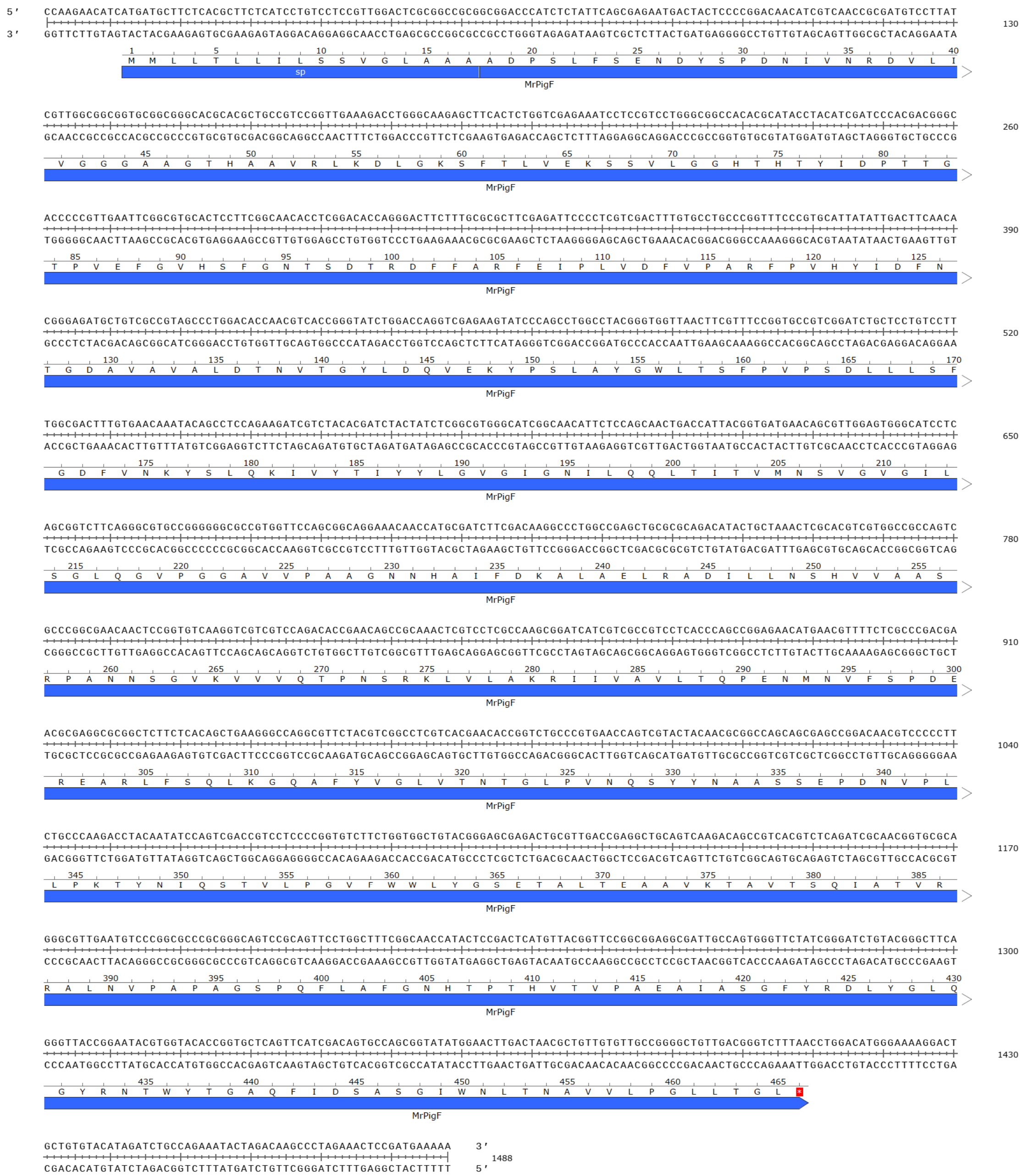

Figure S2 *mrpigF* gene full-length cDNA sequence.
